# Ventilation use in nonmedical settings during COVID-19: Cleaning protocol, maintenance, and recommendations

**DOI:** 10.1177/0748233720967528

**Published:** 2020-11-26

**Authors:** Melanie D Nembhard, D Jeff Burton, Joel M Cohen

**Affiliations:** 1 205740 Cardno ChemRisk, San Francisco, CA, USA; 2 Rocky Mountain Center for Occupational and Environmental Health, University of Utah, Salt Lake City, UT, USA; 3 The Cohen Group, San Mateo, CA, USA

**Keywords:** Ventilation, COVID-19, indoor air quality, nonmedical

## Abstract

Coronavirus disease 2019, otherwise referred to as COVID-19, started in China and quickly became a worldwide pandemic. Beginning in March 2020, nonessential businesses in the United States were closed, and many communities were under shelter-in-place orders. As of May 2020, some business sectors started reopening, even amidst concerns of worker health as the pandemic continued. In addition to physical distancing, cleaning and disinfection routines, and using face coverings, building ventilation can also be an important risk mitigation measure for controlling exposure to SARS-CoV-2 indoors. A number of studies to date, however, have focused on ventilation in medical facilities (e.g. hospitals) as the risk of transmission of SARS-CoV-2 is higher there (because of the close proximity of workers to patients who have the disease and their treatment procedures). Few studies have focused on ventilation use in nonmedical settings (e.g. office buildings and school classrooms), despite the large population of workers and community members in these facilities. In this article, we review the role that building ventilation can play in minimizing the risk of SARS-CoV-2 transmission in nonmedical environments and some recommended protocols to follow for its proper use, including cleaning and maintaining mechanical ventilation systems for businesses, schools, and homes.

## Introduction

At this time, the SARS-CoV-2 virus is thought to be “primarily transmitted between people through respiratory droplets and contact routes” or, more specifically, via fomite transmission, which is human contact “with surfaces in the immediate environment or with objects used on the infected person” (Correia et al., 2020; WHO, 2020a: paras 1 and 2). Sneezes, coughs, and even talking or singing can generate small respiratory droplets that can carry the virus and transmit disease to others. These droplets are emitted into the air, usually straight out or down from the face (if the person does not cough or sneeze into a sleeve or tissue). The SARS-CoV-2 virus is roughly 0.1 µm (100 nm) in diameter (Bar-On et al., 2020). Specifically, droplet transmission is characterized by a person being within 1 m or in “close contact” with someone who has respiratory symptoms (WHO, 2020a: para. 2).

Respiratory droplets (released by respiratory mechanisms) enter the environment in various sizes and are defined as being >5–10 µm in diameter (WHO, 2020a). These larger droplets may rapidly settle out of the air and land on nearby surfaces: floors, tables, clothing, chairs, food, and so forth, potentially contaminating them (Jayaweera et al., 2020). If inhaled through close proximity contact, the larger airborne droplets can be caught in the upper regions of the pulmonary system, for example, in the throat, nose, and upper areas of the lungs, which can result in infection (Jayaweera et al., 2020; WHO, 2020a, 2020b). Smaller droplets (<5 µm), also referred to as droplet nuclei or aerosols, tend to move with the air in which they are floating and can be inhaled and enter the lower reaches of the lung, possibly resulting in infection (Jayaweera et al., 2020). Regarding transmission of these smaller droplets, the Centers for Disease Control and Prevention (CDC) states that “the contribution of small respirable particles to close proximity transmission is currently uncertain. Airborne transmission from person-to-person over long distances is unlikely” (CDC, 2020a: answer #15). These smaller droplets should be considered when discussing transmission routes of SARS-CoV-2 (Jayaweera et al., 2020; Morawska and Cao, 2020; Morawska and Milton, 2020). As of October 5, 2020, the CDC included airborne transmission as another principal route of exposure of respiratory viruses, and specifically noted that “[a]irborne transmission of SARS-CoV-2 virus can occur under special circumstances” (CDC, 2020c: section 7).

In medical settings, aerosol-generating procedures can be commonplace, and therefore risk of infection from airborne transmission is increased. Ventilation systems in medical facilities are in place as a measure to control close proximity (either droplet or contact) transmission (ASHRAE, 2020a; Morawska et al., 2020). According to the World Health Organization (WHO) 2009 guidance for infection control in health-care settings, natural ventilation is widely used and accepted as “among the effective environmental measures to reduce the risk of spread of infections in health-care settings” (Atkinson et al., 2009: xix). However, natural ventilation needs to provide at least a ventilation rate of 160 L/s/patient for facilities that are new and have had major renovations (Atkinson et al., 2009). Additionally, general ward and outpatient departments must have 60 L/s/patient, and in corridors and other spaces, it is 2.5 L/s/m^3^ when an infected patient is not being transferred through those spaces (Atkinson et al., 2009). For nonmedical settings, the WHO and the American Society of Heating, Refrigerating and Air-Conditioning Engineers (ASHRAE) do not provide specific guidance on proper ventilation rates to reduce infection transmission. However, based on a position document on infection aerosols created by ASHRAE, ventilation “designs that achieve higher ventilation rates will reduce risk” (ASHRAE, 2020a: 6). Additionally, ASHRAE provides general ventilation guidance, although it is not specific to the “airborne transmission of airborne viruses, bacteria, and other infectious contagions” (ANSI/ASHRAE, 2019: 20).

While a number of studies to date have focused on ventilation in medical facilities, we found that few studies have focused on ventilation use in nonmedical settings (e.g. office buildings and school classrooms), despite the large population of workers and community members who use these facilities. Thus, in this article, we review best practices for utilizing building ventilation to minimize the risk of SARS-CoV-2 transmission in nonmedical environments, and some recommended protocols to follow for proper use, cleaning and maintaining of mechanical ventilation systems for businesses, schools, and homes.

## Importance of ventilation

Air naturally mixes particulate matter, which includes respiratory particles that are generated from a sneeze, cough, or via someone talking, through the process of dilution and dispersion. Ventilation provides air movement that will assist the dilution and dispersion of smaller particles and is considered to be an effective engineering control.

Multiple national and international public health agencies have reported that improving ventilation in a building can help to reduce the risk of transmission of COVID-19 among individuals (AIHA, 2020; ASHRAE, 2020b; CDC, 2020b; CDC, 2020c; WHO, 2020b). The two types of ventilation include natural and mechanical ventilation. The lower cost and more energy efficient option of ventilation is referred to as natural ventilation (Emmerich et al., 2001). Natural ventilation is the “ventilation provided by thermal, wind or diffusion effects through doors, windows, or other intentional openings in the building” (ANSI/ASHRAE, 2019: 5). Using natural ventilation will increase the amount of outdoor air into the building allowing for dilution of the indoor air (CDC, 2020b). However, natural ventilation can only be effective when the proper environmental conditions and suitable building requirements are available. The second type, mechanical ventilation systems, largely lumped into heating, ventilating, and air conditioning (HVAC) systems, are considered key contributors for regulating good indoor air quality (IAQ), which includes controlling infection transmission. Mechanical ventilation is the “ventilation provided by mechanically powered equipment such as motor-driven fans and blowers but not by devices such as wind-driven turbine ventilators and mechanically operated windows” (ANSI/ASHRAE, 2019: 5).

Within the mechanical ventilation arena, there are three types—exhaust-only, supply-only, and balanced. Exhaust-only ventilation systems provide a fan that pushes indoor air outside of the building and outdoor air is supplied to the building through leaks in the building structure and/or design. Supply-only ventilation systems provide a fan that draws outdoor air into the building, and indoor air is pushed out through exhaust fan ducts. Finally, balanced ventilation systems are a combination of exhaust-only and supply-only ventilation systems, with fans that draw air into and out of the building (Atkinson et al., 2009; US Department of Energy, undated).

In the context of the current COVID-19 pandemic and the potential for future infectious disease outbreaks, using and maintaining the proper ventilation system is more important than ever. Questions regarding the SARS-CoV-2-contaminated aerosols still exist, such as:

- What is the illness transmitting capacity to other people of airborne SARS-CoV-2-contaminated aerosols?
- What particle sizes and concentration can cause illness?
- What happens in a space where several people are contagious or sneezing often?
- How do various conditions and events affect airborne concentration and disease transmission?

Given that ventilation may be a component of reducing the risk of COVID-19 transmission and ongoing risk mitigation measures, we sought to review best practices regarding the proper use, maintenance, and cleaning of ventilation systems in nonmedical settings/buildings.

### Recommendations for using ventilation systems in nonmedical settings

To provide maximum utility of a ventilation system, dilution rate can be increased if the HVAC system is configured to bring outside air into the airflow or by simply opening windows and doors to allow for additional natural ventilation to enter the space. The fresh air exchange will provide greater general dilution of air contaminants throughout the space served by the system. A typical classroom HVAC system, for example, moves the air through the classroom about four times every hour when the system is running. If half of that air is fresh, it can reduce the concentrations of air contaminants in half in about 15 min. To determine the air handling system’s capabilities, the system manual is a great place to look. The manual should also state whether the HVAC system complies with the standards and guidelines outlined by respective local and state codes.

Many airborne particles of concern can be captured or removed by the HVAC filter if the filter is equipped with a good filtration rating. The Minimum Efficiency Reporting Value (MERV) rating on a filter indicates the filter’s efficiency at removing particles from the air as it passes through. The higher the rating, the higher the efficiency. The ratings given are from 1 to 16, and MERV 13 or higher are rated and reported as “efficient at capturing airborne viruses” (ASHRAE, 2020b: section-mechanical air filters). A filter that is widely recognized and used for capturing the smallest particles is the High-Efficiency Particulate Air (HEPA) filter. The HEPA filter “can theoretically remove at least 99.97% of dust pollen, mold, bacteria, and any airborne particles with a size of 0.3 µm” and is preferred to the MERV-16 filters as noted by ASHRAE (ASHRAE, 2020b; USEPA, 2019: para. 1). The Ultra-Low Particulate Air filter can also be used to capture virus particles (Elias and Bar-Yam, 2020). However, all HVAC systems are not made the same. Each system requires the proper size filter and certain filter capacities in order to operate correctly. For places with a furnace, these same conditions also apply. Some older home furnaces cannot handle the more efficient filters because they are too big to fit in the filter housing, or they require too much static pressure to move the air through the filter. If the proper filter sizing, use, and capacity are not included in the system manual, then another place to find this information would be from an HVAC/furnace supplier or installation company. Portable room air HEPA filter units can also help filter the air in bedrooms, offices, classrooms, or other locations where there may be high concentrations of air contaminants (Schoen, 2020).

In addition to a proper filter, appropriate checks should be in place to determine the maximum utility of the system. If there is a specific individual in charge of the HVAC system’s maintenance and operation, then that individual should be asked about the status and capabilities of the system. Questions to ask can include, but are not limited to:

- Is the system running properly?
- What service does it need?
- Are all of its parts clean?
- Does anything need to be done to make it work more effectively?
- Are the plans and specifications available for review, just in case?
- Is there anything else I should know?

Whenever someone is present in a building, make sure that the HVAC system is operating. The system fan should be on, and air should be being moved through the system, even if heating or cooling is not necessary. To meet current standards and codes, modern offices and classrooms have HVAC system controls that keep the fan running at all times or that can be set to a “Fan On” or “Circulate” setting (Burton, 2017). If the HVAC system is not running, check to make sure the fan is set to “On” or “Circulate” so the system is providing a constant airflow at all times that someone is present in the building. The constant air movement will increase local dilution of airborne particles and can remove them at the air filter, particularly when the filter is adequate for collecting the smaller particles. Home furnace fans are not normally on all the time, but can often be set to “On” or “Circulate.” Once the proper setting is on, and the system is checked to make sure the air supply and return grilles are open, a furnace can circulate the air in a house in about 20 min, which can help reduce high concentrations of airborne viral particles in a potentially contaminated room or space. If heating and cooling is being used, then typical buildings have an indoor temperature range of 68°F to 74°F in the winter and between 75°F to 80°F in the summer, as recommended by ASHRAE (NIOSH, 2015).

Make sure to check the supply and return grilles and registers to be sure they are open, operating properly, and that air is flowing through them when the system fan is operating. The outdoor air intakes or controls should be set to the maximum level of fresh outdoor air that the system is capable of handling or providing (depending on the weather, season, operating costs, etc.). System humidifiers also need to be checked to be sure they are clean, maintained, operating properly, and providing an in-room relative humidity of about 40–60%. Individuals can also use a free-standing fan (e.g. pedestal fans, floor fans, wall fans and desk fans) for cooling or to help mix the air in the space, however, having the fan blow from one person directly past another should be avoided.

If odors are present in a space or in the building as a whole, facility management or a consultant should be asked to investigate to determine the source. Typical odor sources might include garbage or trash, rodents or insect nests, stagnant water collected somewhere (e.g. in a wet carpet), rotting plants, spoiled food, mold growth in carpets or walls, dirty ductwork, and dirty kitchens or break rooms. If odors are detected in, or coming from, a space or room where chemicals are stored, determine what the odor source is (e.g. a leaking storage container) and have the problem promptly corrected.

### Maintenance and cleaning of ventilation systems

Through proper maintenance and cleaning, HVAC systems used for various indoor environments can perform at their maximum efficiency. Before cleaning, multiple checks should be completed to ensure the HVAC system is operating at its maximum capacity (Burton, 2020). Some of the checks that can be done include:

- Check the outdoor air intakes to assure that they are clean, open, and not blocked by bushes, defective louvers, and so on.
- Check system filters:
  ○ Are they clean?
  ○ Are they rated at the highest efficiency the system is capable of handling?
  ○ Is there a schedule for filter changes?
- Check and clean air supply and return louvers and registers regularly.
- Check to be sure there are no water leaks or standing water in the building or near the HVAC system, including in the HVAC air inlet plenum.
- Make sure there are no sources of contamination near the air intakes. Reentrainment of exhausted effluents is a common problem that may require engineering changes.

HVAC system cleaning is normally performed by a qualified cleaning company. The work to be done involves removing dirt, slime, mold, debris, and other materials found in either the ductwork or the air handling equipment components (e.g. fans, heating and cooling coils, drain pans, filters, terminal boxes, return air plenums, outdoor air intakes, air mixing locations, etc.) through washing, brushing, vibrating, and vacuum cleaning (Burton, 2020). The cleaning process should follow recognized good practices, which would protect building occupants if they must be present during cleaning (Burton, 2020). However, if individuals are doing the work themselves, then they must be mindful when replacing a dirty filter, as it is better to assume that the filter has viable biologic particles that may include molds, bacteria, and viruses. An individual should follow instructions for filter replacement carefully, such as wearing gloves, respirators and masks, and placing dirty filters in plastic bags (NADCA, 2013; Taylor, 2020). Vacuum cleaning and collection equipment can be located inside or outside of the building. If the equipment must be kept inside, HEPA filtration should be provided for any vacuum discharge that may occur (Burton, 2020).

Ducts and the HVAC system should be kept under negative pressure during the cleaning operation, as doing so lessens the discharge of dirt and dust into the space occupied by residents, workers, and/or individuals within the space (Burton, 2020). If possible, the cleaning of the system should be scheduled for a time when the building is not occupied, and before the first person is to be readmitted, and 10 air changes should be completed in the building to ensure fresh air (Burton, 2020). In the first few weeks, the occupants of the space should be asked if they feel comfortable with the environment, and if they are feeling ill in any way. Any complaints or reported feelings of illness or eye or skin irritation while present in the building should be followed up. The current ANSI/ASHRAE Standard I80-2018, *Standard Practice for Inspection and Maintenance of Commercial Building HVAC Systems*, covers “the minimum acceptable level of maintenance for commercial building HVAC systems” to “provide consistency and improve the thermal comfort, energy efficiency, and indoor air quality of commercial HVAC systems” (ANSI/ASHRAE/ACCA, 2018: p. 2). Therefore, this standard can help determine if system cleaning should be performed (ANSI/ASHRAE/ACCA, 2018).

The National Air Duct Cleaners Association (NADCA) established standard NADCA ACR 2013, Assessment, Cleaning, and Restoration, for “assessing new and existing HVAC systems, evaluating the cleanliness of HVAC system components, and guiding the cleaning and restoration of HVAC systems to a specific level of cleanliness” (NADCA, 2013: p. 2). Using air handling equipment (AHE) cleaning firms that have personnel certified by NADCA and who follow NADCA standards and procedures is recommended (Burton, 2020). It is also important to check your area’s rules for what they require for air duct cleaners; some states require the cleaners to have a mechanical contractor’s license (Burton, 2020).

For consideration of cleaning an HVAC system, a number of questions should be asked of contractors to determine if they are qualified to properly clean the system. These questions include:

- Is the company a member of NADCA? Are their technicians certified through NADCA?
- What protocol will be used for cleaning? Does their protocol allow for protection of the HVAC equipment and the occupants that may be in the space during cleaning?
- Through the cleaning process, can they provide a confirmation of type and quantity of contaminants within the system? Is this confirmation performed through observation or do they need to perform testing?
- Does their process protect against contaminants leaving the system and entering the occupied space during cleaning?
- Are they able to determine where the contaminants may be coming from?
- Is there a way to control the source of contaminants in order to keep the system cleaner and running efficiently longer?
- Will the cleaning performed remove the contaminants effectively?
- Do they only recommend cleaning, or is there another way they propose to get rid of the possible contaminant?
- Are we able to receive a guarantee that the HVAC system is clean once the company is done cleaning?

The answer to all of these questions should be “yes.” Research should be performed to determine if the company has a good reputation, and most importantly, one must ensure that the contractor follows industry-established standards to make certain their HVAC system is getting the best cleaning possible (Burton, 2020).

The ductwork and air pathways of a HVAC system often have insulation for the maintenance of thermal efficiency and to control noise (Burton, 2020). Generally, the insulation is made of fibrous material and is mounted on either the inside or outside of the ductwork or AHE (Burton, 2020). The insulation itself can cause IAQ issues if or when the material frays and fibers and fiber coatings enter the occupied space (Burton, 2020). Additionally, the insulation can also harbor microbiological growths if not maintained correctly (Burton, 2020). Currently, it is believed that wet or contaminated insulation should be removed, and the dry uncontaminated insulation should be cleaned through the use of dry vacuuming in order to minimize the potential of disturbance or damage to the insulation (Burton, 2020). To minimize the cleaning to be performed on the insulation, thermal insulation should be installed on the outside of the duct if possible, the insulation should be kept clean and dry, and the HVAC system filters should be replaced on a manufacturer-approved schedule (Burton, 2020).

Sealants, a product used to coat duct surfaces to prevent the release of dust and dirt particles in the air, are currently not recommended for routine use by the EPA, NADCA, and other organizations that look at duct cleaning protocols (USEPA, 2020a). Currently, there are still many questions on the “safety, effectiveness and overall desirability of sealants,” and the EPA states that sealants “should never be used on wet duct liner, to cover actively growing mold, or to cover debris in the ducts, and should only be applied after cleaning according to NADCA or other appropriate guidelines or standards” (USEPA, 2020a: section 9, question 4).

For sanitization of ductwork, the EPA has a minimal amount of registered products, and they are specifically for “use on the inside of bare sheet metal air ducts,” and there are currently no products that are “registered as biocides for use on fiber glass duct board or fiber glass lined ducts” (USEPA, 2020a: section 9, question 3). Before cleaning, the type of system should be understood to determine the best type of cleaning and the products that should be used during cleaning. To prevent contamination of the HVAC system, follow good housekeeping practices in occupied spaces. Return air plenums and HVAC system components should be kept clean and dry, and the dirt, debris, and microbial growth that occurs in the system should be reduced (Burton, 2020).

Ultraviolet germicidal irradiation (UVGI) has also been noted as a process that can reduce the risk of “dissemination of infectious aerosols in buildings” (ASHRAE, 2020a; CDC, 2020b). According to ASHRAE, this air and surface type of disinfection can be used in nonmedical settings, including residences, commercial properties, and schools (ASHRAE, 2020c). A number of ultraviolet (UV) systems can also be utilized for the ventilation system, such as one used to maintain a clean HVAC system, others to provide “on-the-fly inactivation of microorganisms in moving airstreams,” some to be mounted above building occupants to create an “irradiated zone,” and mobile UV units for cleaning and surface disinfection (ASHRAE, 2020c: answer #6).

ASHRAE’s Position Paper on infectious aerosols from April 2020 states, “[t]ransmission of SARS-CoV-2 through the air is sufficiently likely that airborne exposure to the virus should be controlled” (ASHRAE, 2020a: p. 2). ASHRAE also states, “[v]entilation and filtration provided by heating, ventilating, and air conditioning systems can reduce the airborne concentration of SARS-CoV-2 and thus the risk of transmission through the air” (ASHRAE, 2020a: p. 2).

ASHRAE makes the following recommendation for non-health-care buildings:

– Increase outdoor air ventilation to 100% through opening of outdoor air dampers (should only be done when conditions outdoors permit),
– Use MERV-13 or higher air filtration,
– Keep systems running 24/7, if possible,
– Add portable room HEPA or high-MERV air cleaners,
– Add UVGI devices connected to in-room fans in high-density spaces, for example, waiting rooms,
– Maintain temperature and humidity levels per recommendations regarding the infectious aerosol of concern,
  ○ Several publications have recommended maintaining humidity in the 40–60% range.
  ○ ASHRAE does not recommend an indoor temperature and humidity to control aerosol transmission of COVID-19.
– Bypass energy recovery ventilation systems that potentially leak exhaust air back into the air supply (ASHRAE, 2020a).

Overall, these are good recommendations to follow. In light of the pandemic, and in an abundance of caution, following the above ASHRAE guidelines is highly suggested. Additionally, the American Conference of Governmental Industrial Hygienists (ACGIH) Industrial Ventilation Committee published a white paper that provides “recommended practices” for ventilation systems specific to industrial settings (American Conference of Governmental Industrial Hygienists (ACGIH) Industrial Ventilation Committee, 2020; p. 2).

## Discussion

As COVID-19 transmission continues throughout the world and the public desires a return to normal operations, the protection of the general public is of the utmost importance. With various businesses and schools reopening, ventilation is an important engineering control to limit the potential transmission of SARS-CoV-2. As discussed above, transmission of the SARS-CoV-2 virus occurs by direct or indirect close contact with infected persons and their infected respiratory secretions. While the evidence is still being gathered, multiple national and international public health agencies have reported that improving ventilation in a building can help to reduce transmission of the virus among individuals (AIHA, 2020; ASHRAE, 2020b; CDC, 2020b; CDC, 2020c; WHO, 2020b). Despite these recommendations, a number of researchers have argued that ventilation is not a means of reducing transmission because aerosol airborne transmission has not been proven to be a route of concern (outside of medical settings) except for close proximity to the source of emission. WHO indicated in early July that airborne transmission outside of medical settings needs further study (WHO, 2020b). On the other hand, some current scientific evidence supports the view that airborne aerosol transmission outside of medical settings may be possible. As of August 2020, additional evidence has been found showing that SARS-CoV-2 “can remain airborne for longer times and further distances than originally thought,” thereby making the need for proper ventilation even more important as an effective engineering control measure (REHVA, 2020; USEPA, 2020b: para. 1). Finally, as of October 5, 2020, the CDC determined that airborne transmission of smaller droplets, or aerosols, is a principal route of exposure of respiratory viruses, and specifically noted that “[a]irborne transmission of SARS-CoV-2 virus can occur under special circumstances” (CDC, 2020c: section 7).

### The current debate regarding transmission via ventilation

There are a few papers in the literature that suggest that an aerosol carrying SARS-CoV-2 can be picked up and drawn through the HVAC system and supplied to another room where individuals can become infected; however, these studies seem quite specific to the area being studied.

As reported by a research letter authored by Lu et al. (2020), one family traveled from Wuhan, China, and dined in a restaurant in Guangzhou, China, next to two other families. All tables were situated about 3 ft (about 1 m) apart. Following this restaurant visit, 10 individuals from three separate families, including the family that traveled from Wuhan, were infected with SARS-CoV-2 and developed COVID-19. The infected person sat at Table A, which was situated in between Tables B and C. The authors determined that droplet transmission could not be the only means of exposure and proposed that “strong airflow from the air conditioner could have propagated droplets from table C to table A, then to table B, and then back to table C” (Lu et al., 2020: p. 1629). These authors reported that aerosol transmission may be less of a factor, since no other diners or the wait staff were infected. Additionally, the air conditioner had smear samples collected that were nucleotide negative (Lu et al., 2020). However, following this research letter, Li et al. (2020) performed a simulation to determine the dispersion of droplets that may have been expired by the initial infected patient. The authors reported that the ventilation rate during this dining experience was only 0.75–1.04 L/s per person or 1.6 cubic feet per minute per person (Li et al., 2020; Taylor Engineering, 2020). Based on this research, however, the authors were unable to conclude that long-range airborne transmission of SARS-CoV-2 could occur in an indoor space (Li et al., 2020). They did confirm, however, that “extended short-range aerosol transmission,” or short-range transmission extended by the use of a low ventilation rate, could be possible (Li et al., 2020: p. 15). Overall, the authors concluded that “extended short-range aerosol transmission of the virus is possible in crowded and poorly ventilated enclosures” and determined that “it is crucial to prevent overcrowding and provide good ventilation in buildings and transport cabins for preventing the spread of SARS-CoV-2 and the development of COVID-19” (Li et al., 2020: p. 15).

The concept of potential transmission through the ventilation system has been reported in other studies; however, a number of studies have provided evidence to the contrary. Taylor Engineering (2020), for example, reviewed multiple studies that researched ventilation systems and the risk they may pose to infection transmission and concluded that “none of the ∼80 research reports we reviewed has demonstrated, and few even suggested, air handling unit (AHU) scale transmission, where aerosols emitted from an infected person in one room were drawn through an [Air Handling Unit], and then supplied to another room where they were inhaled resulting in infection for any virus, even those considered likely to have airborne transmission paths, such as influenza and SARS-CoV-1” (Taylor Engineering, 2020: p. 9). For example, one study Taylor references is the Xu et al. (2020) study of the Diamond Princess Cruise Ship that reported 146 COVID-19 cases among the passengers on the 14-day cruise. Xu et al. (2020: p. 2) concludes, “[t]he ship central air conditioning system did not play a role… Most transmission appears to have occurred through close contact and fomites”.

## Conclusion

Given the current debate regarding transmission of small airborne aerosols, ventilation systems should be used and maintained correctly in order to maximize the utility of the system, reduce the risk of indoor airborne infection, and reduce the possibility that the systems themselves may transmit and spread the disease (as has been reported by some authors) (Correia et al., 2020; Elias and Bar-Yam, 2020). Checking the ventilation system to make sure that the system is properly configured, maintained, and cleaned, that proper filters are installed, that everything is working properly, that there is an increase in the amount of outdoor air, and that there is constant air movement can reduce the risk of COVID-19 transmission. However, just having a ventilation system installed in a building is not enough. Other protective measures should be implemented and followed by building occupants. Additional practices, such as wearing gloves and masks, washing hands and clothing, providing regular surface cleaning, and physical distancing, can all reduce the risk of transmitting COVID-19. Through all of these mitigation efforts, from engineering controls (ventilation) to administrative controls (physical distancing) to the wearing of personal protective equipment (gloves, masks, etc.), we can lower the risk of COVID-19 transmission.
